# The Impact of Monoclonal Antibodies on Airway Smooth Muscle Contractility in Asthma: A Systematic Review

**DOI:** 10.3390/biomedicines9091281

**Published:** 2021-09-21

**Authors:** Luigino Calzetta, Marina Aiello, Annalisa Frizzelli, Giuseppina Bertorelli, Beatrice Ludovica Ritondo, Paola Rogliani, Alfredo Chetta

**Affiliations:** 1 Respiratory Disease and Lung Function Unit, Department of Medicine and Surgery, University of Parma, 43126 Parma, Italy; marina.aiello@unipr.it (M.A.); annalisa.frizzelli@unipr.it (A.F.); giuseppina.bertorelli@unipr.it (G.B.); chetta@unipr.it (A.C.); 2 Unit of Respiratory Medicine, Department of Experimental Medicine, University of Rome “Tor Vergata”, 00133 Rome, Italy; beatriceritondo@gmail.com (B.L.R.); paola.rogliani@uniroma2.it (P.R.)

**Keywords:** airway hyperresponsiveness, airway smooth muscle, monoclonal antibodies, severe asthma

## Abstract

Airway hyperresponsiveness (AHR) represents a central pathophysiological hallmark of asthma, with airway smooth muscle (ASM) being the effector tissue implicated in the onset of AHR. ASM also exerts pro-inflammatory and immunomodulatory actions, by secreting a wide range of cytokines and chemokines. In asthma pathogenesis, the overexpression of several type 2 inflammatory mediators including IgE, IL-4, IL-5, IL-13, and TSLP has been associated with ASM hyperreactivity, all of which can be targeted by humanized monoclonal antibodies (mAbs). Therefore, the aim of this review was to systematically assess evidence across the literature on mAbs for the treatment of asthma with respect to their impact on the ASM contractile tone. Omalizumab, mepolizumab, benralizumab, dupilumab, and tezepelumab were found to be effective in modulating the contractility of the ASM and preventing the AHR, but no available studies concerning the impact of reslizumab on the ASM were identified from the literature search. Omalizumab, dupilumab, and tezepelumab can directly modulate the ASM in asthma, by specifically blocking the interaction between IgE, IL-4, and TSLP, and their receptors are located on the surface of ASM cells. Conversely, mepolizumab and benralizumab have prevalently indirect impacts against AHR by targeting eosinophils and other immunomodulatory effector cells promoting inflammatory processes. AHR has been suggested as the main treatable trait towards precision medicine in patients suffering from eosinophilic asthma, therefore, well-designed head-to-head trials are needed to compare the efficacy of those mAbs that directly target ASM contractility specifically against the AHR in severe asthma, namely omalizumab, dupilumab, and tezepelumab.

## 1. Introduction

Asthma is a chronic, heterogeneous, inflammatory airway disorder consisting of a generally variable airflow limitation and several clinical symptoms including chest tightness, cough, wheezing, and shortness of breath [1]. Airway hyperresponsiveness (AHR) represents a central pathophysiological hallmark of asthma, underpinned by predominant inflammatory traits involving different phenotypes and endotypes [2,3]. Since airway smooth muscle (ASM) is the effector tissue that strives to shorten and constricts the bronchial lumen in response to stimuli, it is mainly implicated in the onset of AHR [4]. Thus, traditionally ASM has been considered as the main effector of AHR exclusively for its contractile properties [5]. However, it is now established that ASM may also have proinflammatory and immunomodulatory functions, by secreting a wide range of cytokines and chemokines [6], thus contributing to structural alterations associated with the disease [7].

Particularly, type 2 (T2)-high asthma is a complex endotype associated with high type 2 inflammatory markers including immunoglobulin E (IgE), interleukins (IL)-4, IL-5, and IL-13 [8], and it is characterized by AHR, eosinophilia, and excessive airway mucus production [9,10].

Although inhaled corticosteroids (ICSs) represent the mainstay of asthma management [11] and are clinically effective in most patients [12], 5–10% of the total asthmatic population remain uncontrolled despite using high doses of ICSs and then systemic corticosteroids [13], a condition associated with a high risk of severe exacerbations, hospitalization, and mortality [12,14].

Over the last decade, improved knowledge regarding the complex pathophysiology of asthma has led to the development of new treatment options [15], and the current Global Initiative for Asthma (GINA) recommendations [11] consider patients with severe asthma associated with T2-high phenotypes/endotypes suitable for add-on biologic therapies [2].

In asthma pathogenesis, a number of inflammatory mediators of the T helper 2-dependent reaction play a pivotal role in a complex signaling environment that contributes to AHR: several experimental studies on isolated airway preparations and in vivo animal models have demonstrated an association between enhanced ASM hyperreactivity and overexpression of IgE [16], IL-4 [17,18], IL-5 [19], IL-13 [18,20,21], and TSLP [22,23].

The approved humanized monoclonal antibodies (mAbs) for the treatment of asthma are targeted against IgE (omalizumab), IL-5 (mepolizumab and reslizumab), IL-5 receptor α (IL-5Rα; benralizumab), and IL-4/IL-13 (dupilumab) [24,25,26,27]. Recently, the Biologics License Application (BLA) for the anti-thymic stromal lymphopoietin (TSLP) tezepelumab has been accepted and granted priority review for the treatment of asthma from the US Food and Drug Administration (FDA) [28]. Considering the immunomodulatory effect of these mAbs, it may be assumed that these drugs may have significant direct and indirect effects on ASM. Therefore, the aim of this article was to systematically review the evidence across literature on current mAbs for the treatment of asthma with respect to their impact on ASM contractile tone.

## 2. Methods

### 2.1. Review Question

The question of this systematic review was to assess if current mAbs for the treatment of asthma may have an impact on ASM contractility and AHR.

### 2.2. Search Strategy and Study Eligibility

**Figure 1:**
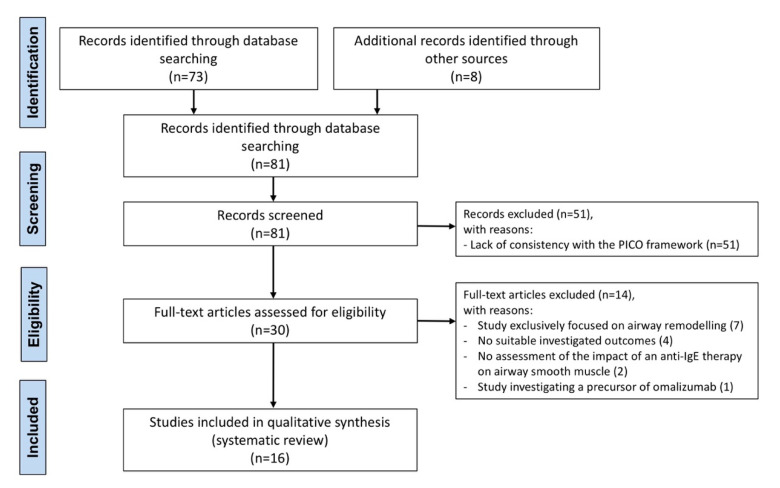
PRISMA flow diagram for the identification of the studies included in the systematic review concerning the impact of mAbs for the treatment of asthma on the airway smooth muscle. IgE: immunoglobulin E; mAbs: monoclonal antibodies; PICO: Patient problem, Intervention, Comparison, and Outcome; PRISMA: Preferred Reporting Items for Systematic Reviews and Meta-Analysis.

The protocol of this synthesis of the current literature has been submitted to the international prospective register of systematic reviews (PROSPERO, submission ID: 270261), and performed in agreement with the Preferred Reporting Items for Systematic Reviews and Meta-Analysis Protocol (PRISMA-P) [29], with the relative flow diagram shown in Figure 1. This study satisfied all the recommended items reported by the PRISMA-P checklist [29].

The PICO (Patient problem, Intervention, Comparison, and Outcome) framework was applied to develop the literature search strategy and question, as previously reported [30]. Namely, the “Patient problem” included asthma; the “Intervention” regarded the administration of mAbs; the “Comparison” was performed with respect to baseline or placebo (PBO); the assessed “Outcome” was the impact on ASM contractility and AHR.

A comprehensive literature search was performed for in vitro, ex vivo, and clinical studies evaluating the impact of current mAbs for asthma on ASM contractility and AHR.

The search was performed on ClinicalTrials.gov (accessed on 15 August 2021), Cochrane Central Register of Controlled Trials (CENTRAL), Embase, EU Clinical Trials Register, MEDLINE, Scopus, and Web of Science in order to provide relevant studies available with no time limit up to 5 July 2021.

The research string was as follows: (“dupilumab”[Supplementary Concept] OR “dupilumab”[All Fields] OR (“tezepelumab”[Supplementary Concept] OR “tezepelumab”[All Fields]) OR (“mepolizumab”[Supplementary Concept] OR “mepolizumab”[All Fields]) OR (“benralizumab”[Supplementary Concept] OR “benralizumab”[All Fields]) OR (“reslizumab”[Supplementary Concept] OR “reslizumab”[All Fields]) OR (“omalizumab”[MeSH Terms] OR “omalizumab”[All Fields] OR “omalizumab s”[All Fields])) AND (((“airway”[All Fields] OR “airway s”[All Fields] OR “airways”[All Fields]) AND (“muscle, smooth”[MeSH Terms] OR (“muscle”[All Fields] AND “smooth”[All Fields]) OR “smooth muscle”[All Fields] OR (“smooth”[All Fields] AND “muscle”[All Fields]))) OR ((“airway”[All Fields] OR “airway s”[All Fields] OR “airways”[All Fields]) AND (“hyperresponsive”[All Fields] OR “hyperresponsiveness”[All Fields] OR “hyperresponsivity”[All Fields])) OR ((“isolate”[All Fields] OR “isolate s”[All Fields] OR “isolated”[All Fields] OR “isolates”[All Fields] OR “isolating”[All Fields] OR “isolation and purification”[MeSH Subheading] OR (“isolation”[All Fields] AND “purification”[All Fields]) OR “isolation and purification”[All Fields] OR “isolation”[All Fields] OR “isolations”[All Fields]) AND (“bronchi”[MeSH Terms] OR “bronchi”[All Fields] OR “bronchus”[All Fields])) OR ((“isolate”[All Fields] OR “isolate s”[All Fields] OR “isolated”[All Fields] OR “isolates”[All Fields] OR “isolating”[All Fields] OR “isolation and purification”[MeSH Subheading] OR (“isolation”[All Fields] AND “purification”[All Fields]) OR “isolation and purification”[All Fields] OR “isolation”[All Fields] OR “isolations”[All Fields]) AND (“airway”[All Fields] OR “airway s”[All Fields] OR “airways”[All Fields]))). Citations of previously published relevant reviews were examined to select further pertinent studies (if any) [31].

Two reviewers independently checked the relevant studies identified from the literature search. The studies were selected in agreement with PICO and any difference in opinion about eligibility was resolved by consensus.

### 2.3. Data Extraction

Data from included studies were extracted in agreement with Data Extraction for Complex Meta-anALysis (DECiMAL) recommendations [32], and checked for study references and characteristics, number and characteristics of the analyzed patients or donors or animals with age and gender, type of analyzed samples, treatments and comparators with doses of medications, smoking habits, forced expiratory volume in 1 s (FEV_1_), and outcome measurements to evaluate the impact on ASM.

### 2.4. Endpoints

The endpoint of this systematic review was to assess the impact of current mAbs for the treatment of asthma on ASM contractility and AHR.

### 2.5. Strategy for Data Analysis

Data from original papers were extracted and reported via qualitative synthesis.

## 3. Results

### 3.1. Study Characteristics

Of the 81 potentially relevant records identified in the initial search, 16 studies were deemed eligible for qualitative analysis (Table 1). Overall, this systematic review included data obtained from ten randomized controlled trials (RCTs) involving patients with different levels of asthma severity [33,34,35,36,37,38,39,40,41,42], five pre-clinical studies [18,25,43,44,45], and one observational study on patients with severe refractory asthma [46]. Among the pre-clinical studies, two were conducted ex vivo on passively sensitized human bronchi [25,45], one was carried out in vitro on human ASM cells (ASMCs) [44], one was performed both ex vivo on human isolated bronchial tissue and in vitro on ASMCs [18], and one was an in vivo murine model of chronic asthma [43]. The investigated mAbs were omalizumab [35,36,38,40,41,43,44,45,46], mepolizumab [25,34,37,39], benralizumab [25], tezepelumab [33,42], and dupilumab [18]. No available studies concerning the impact of reslizumab on the ASM were identified from the literature search.

### 3.2. Omalizumab

Omalizumab is a humanized mAb that blocks the interaction between IgE and high-affinity receptor FcεRI on inflammatory cells, including mast cells and basophils. Omalizumab is the first mAb approved by the European Medicines Agency (EMA) and FDA for the treatment of patients ≥6 years old with persistent severe allergic asthma, high levels of blood IgE, and at least a sensitization to a perennial allergen [49].

Roth et al. [44] performed an in vitro study to investigate the impact of omalizumab administered at 0.1 μg/mL, 0.5 μg/mL, and 1.0 μg/mL on primary human ASMCs isolated from allergic asthma donors and stimulated with IgE 1 μg/mL. Treatment with omalizumab dose-dependently inhibited the IgE-induced overexpression of IL-6 and IL-8, by eliciting a significant (*p* < 0.05) reduction when administered at 0.5 μg/mL and 1.0 μg/mL for IL-6, and at 1 μg/mL for IL-8. Omalizumab also suppressed the IgE-induced synthesis of IL-6 and IL-8 messenger RNAs (mRNAs), with a significant (*p* < 0.05) effect achieved when administered at 1.0 μg/mL [44]. Protein secretion of tumor necrosis factor-alpha (TNF-α) was downregulated by omalizumab as well, with a significantly lower effect on ASMCs from asthmatic donors than on control cells, whereas the reduction in TNF-α mRNA synthesis was not different compared to control [44]. When omalizumab was administered at 1.0 μg/mL, TNF-α release and synthesis were significantly (*p* < 0.05) decreased. Protein and mRNA expression of IL-4 was significantly (*p* < 0.05) reduced with omalizumab 1.0 μg/mL [44]. In ASMCs, the expression of high- and low-affinity IgE receptors was not significantly (*p* > 0.05) modulated by omalizumab, but as suggested by the authors, this could be the result of different cell types and culture environment, or different lengths of the observation period [44].

Kang et al. [43] investigated the effects of anti-IgE therapy on the AHR in a murine model of chronic asthma. The administered mAb was clone R35–92, a purified rat anti-mouse IgE mAb showing efficacy in murine asthma models that parallels the results reported for omalizumab in allergic asthmatic patients [50]. Upon chronic exposure to ovalbumin (OVA), mice developed a sustained AHR to methacholine and showed increased levels of α-smooth muscle actin (α-SMA) [43]. Treatment with anti-IgE therapy significantly (*p* < 0.05) inhibited the AHR [43].

An ex vivo study [45] investigated the impact of omalizumab administered at 60 μg/mL, 80 μg/mL, and 120 μg/mL on specific and nonspecific AHR, in both proximal and distal human airways passively sensitized with serum from asthmatic donors. One-hour treatment with OMA 120 μg/mL significantly (*p* < 0.05) reduced the contractile response to cumulative concentrations of histamine (His) in small airways [45]. Concentration-dependent curves indicated that omalizumab 60 μg/mL and 120 μg/mL maximally inhibited the His-induced AHR in small airways and medium bronchi, respectively [45]. At all concentrations, omalizumab significantly (*p* < 0.05) suppressed the specific contractile response to Dermatophagoides pteronyssinus in both medium bronchi and small airways [45].

In an RCT [41] performed on patients with mild allergic asthma, the anti-IgE mAb rhuMAb-E25 (later named omalizumab) delivered at a dose of 0.5 mg/kg for nine visits did not significantly improve the provocative concentration of methacholine causing a 20% FEV_1_ (PC_20_) compared to PBO. However, 24 h after the second allergen challenge, PC_20_ was significantly (*p* < 0.05) higher (0.45 mg/mL [95%CI 0.06–14.9]) than that on the day after the first challenge, with respect to baseline (0.13 mg/mL) [41].

In the same year, Boulet et al. [40] reported that in patients with mild allergic asthma, the anti-IgE mAb rhuMAb-E25 administered at 1.0 mg/kg during six visits slightly, although significantly (*p* < 0.05), improved methacholine PC_20_.

Prieto et al. [35] conducted an RCT to evaluate the effects of omalizumab administered at 150–300 mg every 4 weeks or at 225, 300, or 375 mg every 2 weeks on bronchoconstriction induced by methacholine and adenosine 5′-monophosphate (AMP) in patients with mild to moderate allergic asthma. In the omalizumab group, the PC_20_ for AMP significantly (*p* < 0.001) increased from 14.32 mg/mL (95%CI 9.72–21.12) to 54.07 mg/mL (95%CI 37.67–79.69) after 4 weeks of treatment and to 53.78 mg/mL (95%CI 36.48–79.28) at 12 weeks [35]. The improvement in AMP PC_20_ was significantly (*p* < 0.05) greater in the patients treated with omalizumab than in those receiving PBO after 4 weeks of treatment, with the mean difference between the two groups being 1.52 doubling concentrations (95%CI 0.25–2.79) [35]. Changes in AMP PC_20_ values were not significantly different between omalizumab and PBO after 12 weeks [35]. Following cessation of treatment, PC_20_ AMP values returned to pretreatment values [35]. The PC_20_ for methacholine increased in the OMA group from 1.27 mg/mL (95%CI 0.82–1.98) to 2.10 mg/mL (95%CI 1.35–3.27) after 12 weeks of treatment and the mean increase doubling the concentrations was not significant [35]. Changes in methacholine PC_20_ values were not significantly different between the OMA and PBO groups, neither when doubling the concentrations [35]. After cessation of OMA, PC_20_ methacholine parameters returned to pretreatment values [35].

A prospective observational study [46] evaluated the efficacy of 48 weeks of treatment with omalizumab on the AHR in patients with severe refractory asthma, despite the use of multiple controller medications. Omalizumab dosing was based on body weight and baseline total serum IgE levels [46]. Omalizumab did not change the airway sensitivity and reactivity to methacholine [46]. According to the authors, these results could have been affected by the limited data coming from computed tomography (CT) and methacholine responsiveness tests, since not all patients could undergo these measurements due to poor lung function and difficulty in breath-holding [46].

After 16 weeks of treatment with omalizumab administered at 150–300 mg every 4 weeks or at 225–375 mg every 2 weeks, AHR to methacholine inhalation was not significantly improved in mild to moderate asthmatic patients with sputum eosinophilia, although at baseline, patients in the PBO group were less responsive to methacholine than the omalizumab group (PC_20_ 0.54 vs. 1.01, respectively) [36]. As stated by the authors of the study, this evidence might not only be indicative of the physiological complexity of AHR, but it might also suggest that IgE and eosinophils, which were significantly decreased with omalizumab, may not mediate methacholine responsiveness in mild to moderate asthma [36].

A sub-study [38] of a previous multicenter RCT [47,48] showed that in patients with moderate to severe allergic asthma, treatment with omalizumab administered at 2- to 4-weeks intervals based on body weight and total IgE at screening, improved the AHR to acetylcholine by inducing a significant (*p* < 0.05) increase in PC_20_ compared to PBO. Three months after completing the therapy, no difference was detected between the treatment groups [38].

### 3.3. Mepolizumab

Mepolizumab is the first humanized anti-IL-5 mAb approved by EMA and FDA for patients ≥6 years old with severe eosinophilic asthma that remains uncontrolled despite GINA step 4 therapy. Mepolizumab targets circulating IL-5 preventing the interaction with the IL-5Rα on the surface of eosinophils, a condition necessary for the development and survival of eosinophils themselves [49].

In passively sensitized medium bronchi, a procedure that reproduces ex vivo the AHR characteristic of asthma, mepolizumab administered at concentrations ≥3 μg/mL significantly (*p* < 0.05) prevented the His-mediated AHR and at ≥10 μg/mL, the ASM contractility was significantly (*p* < 0.05) reduced at the same level of that detectable in control tissues [25]. At least 100 μg/mL of mepolizumab were necessary to significantly (*p* < 0.05) reduce the potency of His (delta negative logarithm of the half-maximal effective concentration [pEC_50_]: 0.65 ± 0.22) [25]. In particular, mepolizumab suppressed the AHR induced by His administered at concentrations eliciting 50%, 70%, and 90% of the maximal effect (EC_50_, EC_70_, and EC_90_; E_max_), by reaching an E_max_ of −108.29 ± 32.16%, and significantly (*p* < 0.05) relaxed the bronchial contractile tone induced by electrical field stimulation (EFS) delivered at frequencies inducing 50%, 70%, and 90% of the maximal effective frequency (EF_50_, EF_70_, and EF_90_), in a concentration-dependent manner [25]. At least 30 μg/mL of mepolizumab was necessary to significantly (*p* < 0.05) reduce the hyperresponsive myogenic tone induced by quick stretch, compared to positive control tissues. Mepolizumab prevented the depletion of cyclic adenosine monophosphate (cAMP) induced by passive sensitization and restored the physiological levels when administered at 10 μg/mL [25]. Interestingly, the inhibition of AHR by mepolizumab was significantly (*p* < 0.05) correlated with the increased cAMP concentrations [25].

Leckie et al. [39] conducted an RCT in mild asthmatic patients to investigate the effects of a single intravenous infusion of SB-240563 (later known as mepolizumab) administered at doses of 2.5 mg/kg or 10.0 mg/kg. Compared to PBO, mepolizumab did not significantly modulate the AHR to His before and after allergen challenges, therefore the authors of the study suggested that blood and sputum eosinophilia might not represent the prerequisite for AHR in relation to allergen exposure and that several other cell types might be involved in these responses [39].

Flood-Page et al. [37] extended the work by Leckie et al. [39] by conducting an RCT on the impact on AHR of three intravenous doses of mepolizumab 750 mg in patients suffering from mild asthma. The study found no significant difference between mepolizumab and PBO groups in reducing the AHR induced by His [37]. According to the authors, this evidence could be explained by the fact that mepolizumab only moderately reduced bronchial tissue eosinophilia and did not modulate the ongoing eosinophilic degranulation within the bronchial mucosa [37].

Haldar et al. [34] conducted an RCT of patients with refractory eosinophilic asthma and a history of recurrent severe exacerbations to assess the impact of mepolizumab 750 mg on the AHR induced by methacholine [34]. After 12 weeks of treatment, mepolizumab did not induce an improvement of the AHR [34].

### 3.4. Benralizumab

Benralizumab is a humanized afucosylated mAb directed against IL-5Rα and not against the circulating IL-5. The complex of mAb/IL-5Rα promotes the apoptosis of eosinophils via antibody-dependent cell-mediated cytotoxicity, a mechanism involving natural killer cells leading to active peripheral eosinophils depletion. Benralizumab is approved by the EMA for adult patients and by the FDA for subjects aged ≥12 years old as an add-on treatment for uncontrolled severe eosinophilic asthma [49].

In passively sensitized human medium bronchi, benralizumab administered at ≥1 μg/mL significantly (*p* < 0.05) reduced the E_max_ elicited by the concentration-response curve to His and when administered at 100 μg/mL, the contractility was reduced to a significantly (*p* < 0.05) lower level than that detectable in the negative control tissues [25]. At concentrations ≥10 μg/mL, benralizumab significantly (*p* < 0.05) reduced the potency of His (pEC_50_: 0.65 ± 0.20) [25]. Specifically, the anti-IL-5Rα mAb suppressed the AHR induced by His administered at EC_50_, EC_70_, and EC_90_, by reaching an E_max_ of −134.14 ± 14.93% [25]. Moreover, benralizumab inhibited the contractile response to EFS delivered at EF_50_, EF_70_, and EF_90_, in a concentration-dependent manner [25]. Benralizumab also suppressed the hyperresponsive myogenic tone induced by quick stretch and when administered at 10 μg/mL, the contractile response was reduced to a level significantly (*p* < 0.05) lower than that detectable with negative controls [25]. Benralizumab restored the physiological levels of cAMP, and interestingly, inhibition of the AHR was significantly (*p* < 0.05) correlated with the increased cAMP concentration [25]. As suggested by the authors, the improvement in the level of cAMP may represent a central mechanism to which the inhibition of the IL-5/IL-5Rα pathway may converge, thus leading to the AHR prevention [25].

### 3.5. Dupilumab

Dupilumab, the last approved mAb for the treatment of asthma, acts against the α chain of IL-4 receptor (IL-4R) that is common to both IL-4R and IL-13R. IL-4 and IL-13 are crucial in type 2 inflammation, leading to AHR [49].

Recently, Manson et al. [18] demonstrated that in human isolated small airways, IL-4 and IL-13 increased the potency of His and pretreatment with dupilumab administered at 1 μM significantly (*p* < 0.05) abolished the responses to both IL-4 (pEC_50_: 6.7 ± 0.1 vs. 6.1 ± 0.2) and IL-13 (pEC_50_: 7.1 ± 0.1 vs. 6.3 ± 0.1). In ASMCs, dupilumab blocked the increase in the E_max_ of His-induced intracellular calcium mobilization caused by IL-13 and IL-4 [18].

### 3.6. Tezepelumab

Tezepelumab, recently accepted and granted priority review from the FDA for the treatment of asthma [28], is a first-in-class anti-TSLP mAb that reduces asthma exacerbations and airway inflammation, and improves pre-bronchodilator FEV_1_ in patients with uncontrolled asthma, regardless of baseline blood eosinophil count [49].

The UPSTREAM RCT [33] investigated the effect of tezepelumab 700 mg on the AHR induced by mannitol in patients with uncontrolled asthma. After 12 weeks of treatment, tezepelumab administered at 700 mg induced a numerical improvement in the provoking dose of mannitol causing a 15% reduction in FEV_1_ (PD_15_) compared to PBO, and a most pronounced effect was detected in subjects affected by eosinophilic asthma [33]. At the end of the treatment period, the proportion of patients without AHR to mannitol was significantly (*p* < 0.05) higher in the tezepelumab group than in the PBO group (nine vs. three) [33].

Similarly, the very recent CASCADE RCT [42] demonstrated that in patients with uncontrolled moderate to severe asthma, tezepelumab 210 mg administered for 28 weeks significantly (*p* < 0.05) reduced the AHR to mannitol vs. PBO, both in terms of absolute change in PD_15_ (197.4 mg [95% CI 107.9–286.9] vs. 58.6 mg [95% CI −30.1–147.33], respectively), and in doubling dose units (1.41 mg [95% CI 0.84–1.99] vs. 0.57 mg [95% CI 0.01–1.13], respectively). A numerically higher proportion of patients in the tezepelumab group showed a negative mannitol test compared to PCB at the end of the treatment period (13 [43.0%] vs. 7 [25%]) [42].

## 4. Discussion

**Figure 2:**
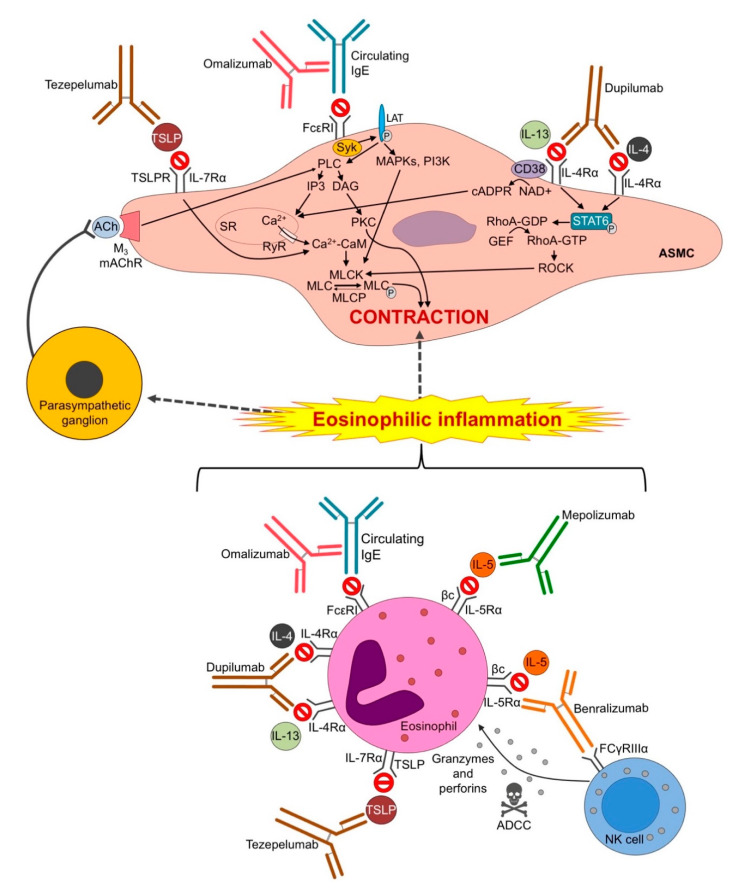
Direct and indirect mechanisms of action against AHR of the mAbs resulting from this systematic review. Parasympathetic ganglia are innervated by preganglionic nerve fibers carried by the vagus nerves and they are found in abundance throughout the walls of medium bronchi. ACh: acetylcholine; ADCC: antibody-dependent cell cytotoxicity; ASMC: airway smooth muscle cell; Ca^2+^: calcium; CaDPR: Cyclic adenosine 5′-diphosphate ribose; CaM: calmodulin; DAG: diacylglycerol; FDA: Food and Drug Administration; GDP: guanosine diphosphate; GEF: guanine nucleotide-exchange factor; IgE: immunoglobulin E; IL-n: interleukin-n; IL-nR: interleukin-n receptor; IP3: inositol trisphosphate; LAT: linker for T-cell activation; mAbs: monoclonal antibodies; MAPK: mitogen-activated protein kinase; mAChR: muscarinic acetylcholine receptor; MLC: Myosin light chain; MLCK: myosin light chain kinase; MLCP: Myosin light chain phosphatase; NAD+: Nicotinamide adenine dinucleotide; NK: natural killer; PKC: protein kinase C; PLC: phospholipase C; RhoA: Ras homolog family member A; ROCK: Rho-associated protein kinase; RyR: ryanodine receptor; SR: sarcoplasmic reticulum; STAT6: signal transducer and activator of transcription 6; TSLP: thymic stromal lymphopoietin; TSPLR: TSLP receptor.

The evidence resulting from this systematic review indicates that omalizumab, dupilumab, and tezepelumab may modulate AHR via direct action on ASMCs and indirect influence on eosinophilic inflammation and parasympathetic activity; conversely, mepolizumab and benralizumab seem to prevent AHR prevalently by reducing airway inflammation and vagal firing (Figure 2).

ASMCs are capable of expressing the heterodimeric TSLP receptor (TSLPR), consisting of the IL-7 receptor-α chain (IL-7Rα) and TSLPR subunit [22], the heterodimeric IL-4 and IL-13 receptor (IL-4R and IL-13R) complexes which share the common subunit IL-4Rα [51], and the high-affinity receptor for IgE, also known as FcεRI [52]. By contrast, the heterodimeric IL-5 receptor (IL-5R), consisting of an α subunit specific only to IL-5 binding and the common receptor β subunit (βc), is not present on the surface of ASMCs [53].

Eosinophils, which are central to the pathogenesis of allergic and non-allergic asthma [54,55], express high levels of surface IL-5Rα chain [56], as well as TSLPR [57], the IL-4Rα chain common to IL-4R and IL-13R [58,59], and the high-affinity receptor FcεRI [60]. In addition, further inflammatory cells, including dendritic cells, lung epithelial cells, lymphocytes, mast cells, monocytes, stromal cells, and type 2 innate lymphoid cells have been shown to express the heterodimers IL-4Rα/IL-13Rα1 [61,62] and TSLPR [63,64,65], while basophils and mast cells represent the two main cell populations expressing IL-5R and FcεRI [66,67].

Therefore, the anti-IgE omalizumab, the anti-IL-4/IL-13 dupilumab, and the anti-TSLP tezepelumab are supposed to have the ability to directly modulate the ASM contractility and AHR in asthma, by specifically blocking the interaction between the ligands and FcεRI, IL-4Rα, and TSLPR respectively, and indirectly by targeting eosinophils and further inflammatory cells, thus leading to a reduction in the inflammatory cascade.

Conversely, mepolizumab and reslizumab, which selectively block circulating IL-5, and benralizumab, which prevents the adhesion of IL-5 to the IL-5Rα chain, may indirectly modulate the contractile properties of ASMCs by targeting eosinophils and other immunomodulatory effector cells responsible for promoting inflammatory processes of asthma.

The direct action of omalizumab, dupilumab, and tezepelumab on the ASM is mediated by complex intracellular signaling pathways (Figure 2). Omalizumab is known to recognize and sequester free serum IgE, thereby causing the high-affinity receptor FcεRI to be down-regulated [68]. In the absence of omalizumab, the FcεRI signaling cascade requires crosslinking of multivalent antigen bound to IgE, leading to the activation of Syk kinase [69]. Prevalent notion suggests that Lyn kinase is necessary to phosphorylate immunoreceptor tyrosine activating motifs (ITAMs) on FcεRI in order to provide a docking site for activated Syk, however, evidence from electron microscopy and biochemical investigations showed that Lyn dissociates from FcεRI as soon as the latter is stimulated [70,71]. Activated Syk mediates the phosphorylation of the linker for activation of T cells (LAT) that in turn activates phospholipase C (PLC) [69]. PLC catalyzes the hydrolysis of phosphatidylinositol 4,5-bisphosphate (PIP-2) to inositol-1,4-5-triphosphate (IP3) and diacylglycerol (DAG) [72], and in turn IP3 mediates calcium mobilization from the sarcoplasmic reticulum (SR) [73]. In ASMCs, the fast and transient increase in intracellular calcium activates the calcium/calmodulin-sensitive myosin light chain (MLC) kinase (MLCK), followed by phosphorylation of the regulatory MLC and subsequent ASM contraction [74]. Interestingly, evidence that the IgE-induced MLCK expression is also mediated by multiple signaling pathways including mitogen-activated protein kinases (MAPKs) and phosphatidylinositol 3-kinase (PI3K) was provided by Balhara et al. [75].

Dupilumab prevents IL-4 and IL-13 signaling by blocking the IL-4Rα chain common to the IL-4R and IL-13R complexes [76]. Recent evidence has demonstrated that both cytokines directly contribute to the development of AHR at the level of ASMCs [18]. IL-4 and IL-13 upregulate RhoA-GDP by inducing the phosphorylation of signal transducer and activator of transcription 6 (STAT6) [21,77]. RhoA-GDP is activated by Rho guanosine nucleotide exchange factor (GEF), which promotes the exchange of GDP for GTP and the active RhoA-GTP stimulates Rho kinase (ROCK) to inhibit MLCP [78,79] thereby triggering ASM contraction. Another mechanism by which IL-13 is able to modulate ASM contractility has been postulated by Deshpande et al. [80,81]: IL-13 increases the expression of CD38, a cell surface hydrolase and cyclase highly expressed in asthmatic ASMCs, which catalyzes the production of cyclic adenosine diphosphate-ribosyl cyclase (cADPR), an activator of the ryanodine receptor (RyR).

Tezepelumab selectively blocks human TSLP from interacting with the heterodimeric TSLPR complex [82]. Compared with healthy controls, TSLP is highly expressed in the airways of asthmatic patients [83,84]. In vitro evidence has demonstrated that TSLP stimulates ASMCs to enhance intracellular calcium responses to contractile agonists, thus suggesting TSLP to be a potential mediator of ASM contractility [22].

Only recently, an ex vivo study provided evidence that targeting the IL-5/IL-5R pathway is effective against AHR in passively sensitized human medium bronchi [25]. Passive airway sensitization reproduces ex vivo an IgE-dependent AHR in presence of further serum factors and promotes the adhesion of eosinophils to parasympathetic nerves, thereby stimulating their degranulation with subsequent major basic protein (MBP) release. MBP causes the loss of function of the M_2_ muscarinic acetylcholine (ACh) receptor (mAChR) present on postganglionic parasympathetic nerves, resulting in an excessive ACh release [85]. It has been suggested that benralizumab and mepolizumab prevent the AHR to His and EFS by indirectly inhibiting endogenously released intermediaries of bronchoconstriction [25]. Indeed, His indirectly promotes the release of ACh from parasympathetic nerves [86], while EFS mediates the sensitization of vagal parasympathetic fibers causing the release of ACh [87,88] The interaction between ACh and M_3_ mAChRs expressed on post-junctional ASMCs stimulates PLC to produce diacylglycerol (DAG), which in turn activates phosphokinase C to promote ASM contraction [89].

The present systematic review supports evidence for the direct efficacy of omalizumab, dupilumab, and tezepelumab, and the indirect beneficial effect of mepolizumab and benralizumab in modulating the contractility of the ASM and preventing AHR. Omalizumab generally improved AHR in vitro and in vivo, although surprisingly, several RCTs [35,36,41] conducted on mild to moderate asthmatic patients and one observational study [46] on severe refractory asthma reported no beneficial effect of omalizumab against AHR. Nevertheless, these clinical studies were performed on strictly selected and limited groups of patients, therefore these results should be taken with caution.

In vitro and ex vivo evidence indicates that dupilumab effectively reduced the AHR to His [18], whereas tezepelumab improved the ASM contractile response to mannitol in an RCT [33]. Furthermore, both benralizumab and mepolizumab prevented the AHR to His, parasympathetic activation, and mechanical stress, an effect correlated with improved levels of cAMP in hyperresponsive airways [25]. Results from RCTs conducted on patients with mild asthma [37,39] and refractory eosinophilic asthma [34] reported that mepolizumab did not prevent AHR induced by His and methacholine, but the stringent inclusion criteria of the studies might not be representative of broader asthmatic populations.

Interestingly, several studies conducted in vitro on ASMCs, in experimental animal models of asthma or in vivo in humans confirmed the beneficial role of omalizumab [43,46,90,91,92,93,94,95], benralizumab [53], and mepolizumab [34] in reversing airway remodeling. Specifically, OMA reduced collagen and fibronectin deposition in ASMCs [91,92], down-regulated ASM-associated components, mainly myosins and actins [93,95], and reduced the proliferation of ASMCs [92]. In severe asthmatics, omalizumab improved airway wall thickness and the luminal area at the right apical segmental bronchus [46,90] and reduced the number of circulating fibrocytes, which act as precursors of bronchial myofibroblasts [94]. Benralizumab reduced the number of tissue myofibroblasts and the ASM mass [53], while mepolizumab reduced airway wall thickness and total wall area [34].

AHR has been suggested to be a main treatable trait towards precision medicine in patients suffering from eosinophilic asthma [96,97], and together with the post-bronchodilator reversibility test, it is recommended as a tool in asthma diagnosis, classification severity, and control monitoring [98]. Appropriately designed head-to-head RCTs are needed to compare the efficacy of those mAbs directly targeting ASM contractility specifically against AHR in severe asthma, namely omalizumab, dupilumab, and tezepelumab.

Among the currently available mAbs, omalizumab has the greatest amount of data concerning the impact on ASM contractility, as it was first, and for a long time the only available add-on biologic therapy for the treatment of severe asthma [99]. To the best of our knowledge, no study covered any aspect of AHR and ASM contractility in therapy with the currently approved mAb reslizumab.

The limitations of this systematic review are related to the intrinsic characteristics of the included studies: in vitro and ex vivo studies are based on models of asthma, and RCTs have been generally carried out in small and selected populations of asthmatic patients.

In conclusion, the development of biological therapies has led to a significant step forward in the treatment of severe asthma; nevertheless, to date little is still known about the real clinical impact of mAbs against AHR. Future research of currently available and upcoming mAbs for the treatment of severe asthma should address this clinical issue as an important feature of long-term disease management.

## Figures and Tables

**Table 1:** Main characteristics of the studies included in the systematic review.

Study, Year, and Reference	Study Characteristics	Treatment Duration	Type of Cells, Animals, Donors, or Analyzed Patients	Number of Animals, Donors, or Patients	Drugs, Doses and Regimen of Administration	Mean Age (Years)	Male (%)	Current Smokers (%)	Smoking History (Pack-Years)	Post-Bronchodilator FEV_1_ (% Predicted)	Investigated Outcome
Diver et al., 2021 (CASCADE trial) [42]	RCT	Planned 28 wks	Patients with uncontrolled moderate to severe asthma	116	Tezepelumab (210 mg, every 4 wks; SC injection)	50.4	44.0	0.0	NA	69.1	AHR to mannitol
Sverrild et al., 2021 (UPSTREAM trial) [33]	RCT	12 wks	Patients with uncontrolled asthma	40	Tezepelumab (700 mg, every 4 wks; IV infusion)	41.0	42.0	0.0	NA	88.7	AHR to mannitol
Calzetta et al., 2020 [25]	Ex vivo, prospective, randomized, negative- and positive- controlled, blind, parallel-group study	Overnight	Passively sensitized subsegmental bronchi (4–6 mm) from patients undergoing lobectomy for lung cancer (with normal serum IgE levels < 100 IU/mL and normal preoperative lung function parameters)	16	Benralizumab (1 μg/mL–100 μg/mL) vs. mepolizumab (1 μg/mL–100 μg/mL)	50.0	50.0	25.0	24.4	93.1	AHR to His, EFS, and QS and assessment of treatment effect on cAMP levels
Manson et al., 2020 [18]	Ex vivo and in vitro study	1 day	Human small airways and primary ASMCs	33	Dupilumab (1 μM)	69.0	27.0	36.4	NA	NA	AHR to His and EFS
Tajiri et al., 2014 [46]	Prospective, single-arm, observational study	48 wks	Patients with severe refractory asthma	31	OMA (every 2–4 wks, dosing based on body weight and baseline total serum IgE; SC injection)	55.0	32.3	0.0	≤10	93.5	AHR to methacholine
Kang et al., 2010 [43]	In vivo study	3 months	BALB/c mice challenged with OVA (murine model of chronic asthma)	10–15 per group	Rat anti-mouse IgE mAb clone R35–92 (100 μg/200 μL in normal saline), once a month from day 38; IV injection)	8–10 wks	0.0	NA	NA	NA	AHR to methacholine
Roth et al., 2010 [44]	In vitro study	24 h	Primary ASMCs isolated from allergic asthma donors	6	OMA (0.1, 0.5, 1.0 μg/mL)	33.3	66.7	NA	NA	69.0	IL-4, IL-6, IL-8, and TNF-α secretion and synthesis by ASMCs and IgE receptor expression in ASMCs
Haldar et al., 2009 [34]	RCT	1 year	Patients with refractory eosinophilic asthma and a history of recurrent exacerbations	61	Mepolizumab (750 mg every month; IV infusion) vs. PBO	49.0	52.5	0.0	NA	77.9	AHR to methacholine
Berger et al., 2007 [45]	Ex vivo study	1 h	Passively sensitized medium bronchi and small airways obtained from non-atopic and non-asthmatic patients	10	OMA (60, 120, 180 μg/mL)	64.4	100.0	NA	24.5	86.9	ASM contractile responses to His and Dermatophagoides pteronyssinus
Prieto et al., 2006 [35]	RCT	12 wks	Patients with mild to moderate allergic asthma	34	OMA (150–300 mg every 4 wks or 225–375 mg every 2 wks; SC injection) vs. PBO	31.0	47.1	0.0	NA	100.7	AHR to AMP
Djukanovic et al., 2004 [36]	RCT	4 months	Patients with mild to moderate asthma	45	OMA (150–300 mg every 4 wks or 225–375 mg every 2 wks; SC injection) vs. PBO	26.0	46.0	0.0	NA	85.0	AHR to methacholine
Flood-Page et al., 2003 [37]	RCT on	20 wks	Patients with mild asthma	24	Mepolizumab (750 mg, 3 doses; IV infusion) vs. PBO	30.5	70.8	0.0	NA	83.5	AHR to His
Noga et al., 2003 [38]	Sub-study conducted as part of a large multicentre RCT [47,48]	16 wks	Patients with moderate to severe allergic asthma	35	OMA (at least 0.016 mg/kg/IgE IU/mL, every 4 wks; SC injection) vs. PBO	54.3	36.5	NA	NA	79.5	AHR to acetylcholine
Leckie et al., 2000 [39]	RCT	1 day	Patients with mild allergic asthma	24	SB-240563 (2.5 or 10.0 mg/kg, single dose; IV infusion) vs. PBO	27.9	100.0	0.0	NA	88.4	AHR to His
Boulet et al., 1997 [40]	RCT	10 wks	Patients with mild allergic asthma	20	rhuMAb-E25 (1.0 mg/kg; IV infusion) vs. PBO	27.0	60.0	0.0	≤10	92.2	AHR to methacholine
Fahy et al., 1997 [41]	RCT	9 wks	Patients with mild allergic asthma	18	rhuMAb-E25 (0.5 mg/kg; IV infusion) vs. PBO	31.5	NA	NA	NA	94.5	AHR to methacholine

AHR: airway hyperresponsiveness; AMP: adenosine monophosphate; ASMC: airway smooth muscle cell; cAMP: cyclic adenosine monophosphate; CT: computed tomography; EFS: electrical filed stimulation; FEV_1_: forced expiratory volume in the 1st second; His: histamine; IgE: immunoglobulin E; IL-: interleukin-; IV: intravenous; NA: not available; OMA: omalizumab; OVA: ovalbumin; PBO: placebo; QS: quick stretch; RCT: randomized controlled trial; SC: subcutaneous; TNF-α: tumor necrosis factor-alpha; wks: weeks.

## Data Availability

No new data were created or analyzed in this study. Data sharing is not applicable to this article.
